# Aberrant PTEN, PIK3CA, pMAPK, and TP53 expression in human scalp and face angiosarcoma

**DOI:** 10.1097/MD.0000000000026779

**Published:** 2021-07-30

**Authors:** Huiying Wan, Dingding Zhang, Weimin Hu, Zhen Xie, Qiu Du, Qiongrong Xia, Taishen Wen, Haiping Jia

**Affiliations:** a Department of Dermatology, Sichuan Provincial People's Hospital, University of Electronic Science and Technology of China, Chengdu, Sichuan, China; b Department of Medicine, Sichuan Provincial People's Hospital, University of Electronic Science and Technology of China, Chengdu, Sichuan, China; c Department of Immunology and Microbiology, North Sichuan Medical College, Nanchong, Sichuan, China; d Department of Immunology, College of Medical Technology, Chengdu University of Traditional Chinese Medicine, Chengdu, Sichuan, China.

**Keywords:** angiosarcoma, apoptosis, phosphatase and tensin homolog, phosphatidylinositol-4,5-bisphosphate 3-kinase catalytic subunit alpha, phosphorylated mitogen-activated kinase-like protein, tumor protein p53

## Abstract

Angiosarcoma is a rare, highly aggressive malignant tumor originating from endothelial cells that line the lumen of blood or lymphatic vessels. The molecular mechanisms of scalp and face angiosarcoma still need to be elucidated. This study aimed to investigate the expression of phosphatase and tensin homolog (PTEN), phosphatidylinositol-4,5-bisphosphate 3-kinase catalytic subunit alpha (PIK3CA), phosphorylated mitogen-activated kinase-like protein (pMAPK), and tumor protein p53 (TP53) in scalp and face angiosarcoma and to assess tumor tissue apoptosis.

The expression and intracellular distribution of PTEN, PIK3CA, pMAPK, and TP53 proteins in 21 specimens of human scalp and face angiosarcoma and 16 specimens of human benign hemangioma were evaluated using immunohistochemistry. Tumor cell apoptosis was assessed by terminal deoxyribonucleotide transferase-mediated dUTP nick end-labeling staining.

Significantly lower PTEN but higher PIK3CA, pMAPK, and TP53 immunostaining were detected in the angiosarcoma specimens than in the benign hemangioma specimens(*P* < .01). The angiosarcoma tissues exhibited significantly higher apoptosis indices than the benign hemangioma tissues (*P* < .01). The positive expression rates of PIK3CA, pMAPK, and TP53 were correlated with the degree of tumor differentiation in the human scalp and face angiosarcoma.

The PI3K, MAPK, and TP53 pathways might be involved in angiosarcoma tumorigenesis in humans and may serve as therapeutic targets for the effective treatment of this malignancy.

## 1 Introduction

Angiosarcoma is a rare, highly aggressive malignant tumor originating from endothelial cells that line the lumen of blood or lymphatic vessels. Angiosarcoma represents approximately 1% to 2% of soft-tissue sarcomas in humans,^[1]^ and it commonly involves the skin, breast, liver, and distal extremities.^[2]^ Cutaneous angiosarcoma frequently occurs on the scalp and face, especially in elderly men.^[3]^ The lesions may present as bruise-like plaques with irregular edges and can be mistaken for benign lesions, such as hemangiomas.^[4]^ The standard treatment options include surgery, radiotherapy, and chemotherapy. Because angiosarcoma cells are carried by the blood and lymphatic flows, local recurrence and early metastasis are common, and the 5-year survival rate is only approximately 40%, with a median of 16 months.^[5]^ Due to the rarity of this disease, its molecular pathology is not fully understood.

Genomic screening has detected mutations in the tumor protein p53 (TP53),^[6–9]^ PI3K,^[6]^ and mitogen-activated kinase-like protein (MAPK)^[10]^ pathways in angiosarcoma. Immunohistochemistry and functional analysis have identified molecules such as VEGF,^[11]^ survivin,^[12]^ and HSP90 ^[13]^ as potential contributing factors in angiosarcoma pathology. Apoptosis, or programmed cell death, plays a critical role in tissue homeostasis and human diseases. A hallmark of carcinogenesis is the ability of cancer cells to evade apoptosis and continuously proliferate despite their abnormalities.^[14]^The PI3K/Akt and MAPK signaling pathways drive carcinogenesis through their pro-proliferation and anti-apoptotic functions.^[15,16]^ phosphatidylinositol-4,5-bisphosphate 3-kinase catalytic subunit alpha (PIK3CA) is a class I PI3K catalytic subunit. PTEN is a well-established, major negative regulator of the PI3K/Akt pathway. Activating mutations of PIK3CA and inactivating mutations in PTEN are frequently found in human cancers.^[17,18]^ Metabolic and epigenetic aberrations of the MAPK pathway are also prominently associated with carcinogenesis.^[19]^ TP53, a crucial tumor suppressor, is the most frequently mutated gene (>50%) in human cancers.^[20]^ However, the expression of these key cancer-related genes in the scalp and facial angiosarcoma is not clear.

In this study, we investigated the expression of PIK3CA, PTEN, p-MAPK, and TP53 proteins in scalp and face angiosarcoma specimens. We also evaluated tumor tissue apoptosis and discussed the possible involvement of the PI3K, MAPK, and TP53 pathways in the pathogenesis of this rare malignancy.

## 2 Materials and methods

### 2.1 Tumor tissue collection

Skin lesion specimens were collected from 21 patients (13 men and 8 women) diagnosed with scalp and face angiosarcoma at the Department of Pathology, Sichuan Provincial People's Hospital, from January 2000 to December 2019. The patients were 53 to 84 years old, with an average age of 69.2 years. Angiosarcoma was confirmed by a histological examination conducted by 2 experienced pathologists. Sixteen skin hemangioma specimens were used as controls. The study was approved by the Ethics Committee of Sichuan Provincial People's Hospital. Written informed consent was obtained from all the patients. All tissue samples were sectioned using a Leica Microtome (Leica Microsystems, Germany), fixed in formalin, and embedded in paraffin.

### 2.2 Immunohistochemical analysis

After deparaffinization, rehydration, and antigen retrieval in citrate buffer, the slides were treated with 3% hydrogen peroxide in methanol for 20 min to block endogenous peroxidase. The slides were incubated overnight at 4°C with a rabbit polyclonal antibody against human PTEN, PIK3CA, or pMAPK (1:100; Wuhan Boster Biological Technology, Ltd., Wuhan, China), or a mouse monoclonal antibody against human TP53 (1:100; MXB Biotechnologies, Fuzhou, China). After washing with phosphate-buffered saline, the slides were incubated with horseradish peroxidase-conjugated secondary antibodies and visualized using 3,3-diaminobenzidine. The slides were stained with hematoxylin, dehydrated through a graded series of ethanol solutions, sealed, and subjected to microscopic examination. The percentage of positively stained cells was calculated from 10 randomly selected fields in each section (×400). Immunoreactivity was semi-quantitatively graded based on staining intensity and percentage of positively stained cells. The staining intensity was scored from 0 to 3: 0, negative; 1, light yellow; 2, deep yellow; and 3, brownish-yellow. The percentage of positively stained cells was also scored from 0 to 3: 0, ≤5%; 1, 6%–25%; 2, 26%–50%; and 3, >50%. The total score was calculated as the sum of the intensity score and percentage positive score. Finally, immunoreactivity was graded according to the total score as follows: negative (−), ≤1; low (+), 2-3; medium (++), 4–6; and high (+++), >6. Image analysis was performed by 2 experienced pathologists using a computer-aided image analysis system.

### 2.3 TUNEL staining

Cell apoptosis was evaluated using a transferase-mediated dUTP nick end-labeling (TUNEL) staining kit from Roche (USA). Briefly, the tissue sections were deparaffinized, treated with 3% hydrogen peroxide in methanol to inactivate endogenous peroxidases, and digested in proteinase K solution. After repeated washing in distilled water, the slides were subjected to TUNEL staining, according to the manufacturer's instructions. The slides were stained with hematoxylin, dehydrated through a graded series of ethanol solutions, sealed, and subjected to microscopic examination. Apoptotic nuclei were stained in deep yellow. The apoptosis index (AI) was calculated as the average percentage of apoptotic cells from 10 randomly selected fields on each slide (×400).

### 2.4 Statistical analysis

All data were analyzed using SPSS 18.0. Differences in PTEN, PIK3CA, pMAPK, or TP53 immunostaining between the angiosarcoma and hemangioma groups were analyzed using the chi-square test. The correlation between PIK3CA, pMAPK, and TP53 and tumor differentiation was analyzed using Fisher exact test. Differences in cell apoptosis between the 2 groups were analyzed using the *t* test. Statistical significance was set at *P* < .05.

## 3 Results

### 3.1 Clinical characteristics of the patients with scalp and face angiosarcoma

**Figure 1:**
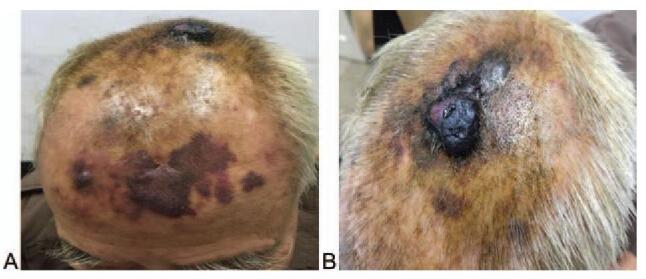
Representative images of angiosarcoma lesions of the scalp. Angiosarcoma lesions can present as a bruise-like lesions and black bumps.

The clinical characteristics of patients with angiosarcoma of the scalp and face, whose tumor specimens were included in this study, are summarized in Table 1. Representative images of angiosarcoma lesions are shown in Figure 1.

**Table 1:** Clinical characteristics of the patients with angiosarcoma of the scalp and face.

Code	Age (year)	Gender	Tumor size (cm)	Tumor location	Treatment	Survival (month)
1	53	Female	4 × 3	Right cheek	Surgery	24^+^
2	73	Male	8 × 6	Left forehead	Surgery/chemo	14
3	62	Female	1.5 × 1.2	Front of the left ear	Surgery	29
4	84	Female	12 × 9	Right temporal	No	3
5	78	Female	3.5 × 3	Left forehead	No	Unknown
6	60	Male	1.8 × 1.5	Top left	Surgery	17
7	55	Female	2 × 1	Center forehead	Surgery	9^+^
8	67	Male	2.5 × 2	Top right	Surgery/radiation	12^+^
9	68	Male	4 × 4	Forehead	No	Unknown
10	75	Female	7 × 5.5	right Forehead	surgery	10
11	68	Female	3 × 2	behind Right ear	Surgery/radiation	5^+^
12	77	Male	5.5 × 4	Top right	Unknown	Unknown
13	69	Male	4 × 3.5	Left forehead	Surgery	Unknown
14	62	Male	3 × 2.5	Left forehead	Surgery/chemo	15^+^
15	82	Female	7 × 7	Right temporal	Radiation	7
16	61	Female	1.5 × 1	Upper eyelid	Surgery	11^+^
17	80	Male	6 × 5	Top left	Surgery	7
18	76	Male	8 × 6	right Forehead	no	5
19	77	Male	4 × 3	left Temporal	Surgery/radiation	19
20	65	Female	2.5 × 2	left Temporal	Surgery	Unknown
21	62	Female	8 × 5	top	Surgery	6^+^

### 3.2 PTEN was downregulated in scalp and face angiosarcoma

**Figure 2:**
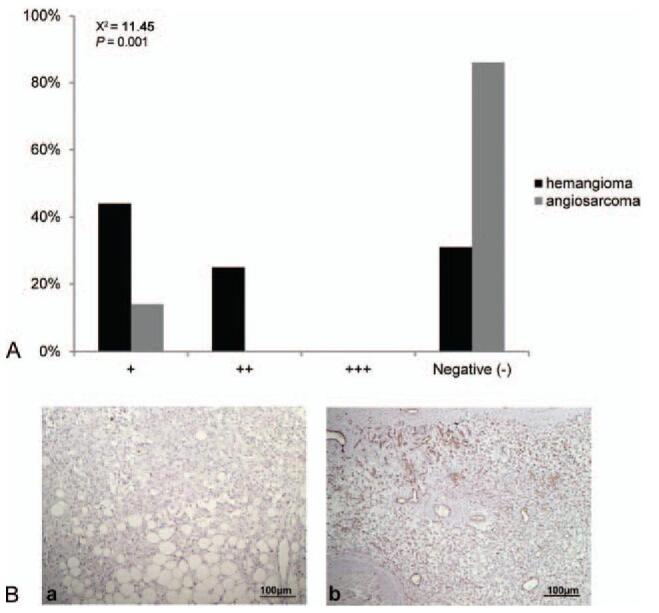
(A) PTEN immunoreactivity in angiosarcoma of the scalp and face and hemangioma. Angiosarcoma lesions exhibited significantly weaker PTEN immunoreactivity than hemangioma (χ^2^ = 11.45, *P* = .001, *P* < .01). (B) Representative images of immunohistochemical staining of PTEN in (a) angiosarcoma and (b) hemangioma tissues (SP, ×100). PTEN = phosphatase and tensin homolog.

PTEN was mostly detected in the cytoplasm. Angiosarcoma lesions exhibited significantly weaker PTEN immunoreactivity than hemangiomas (χ^2^ = 11.45, *P* = .001, Fig. 2).

### 3.3 PIK3CA was upregulated in scalp and face angiosarcoma

**Figure 3:**
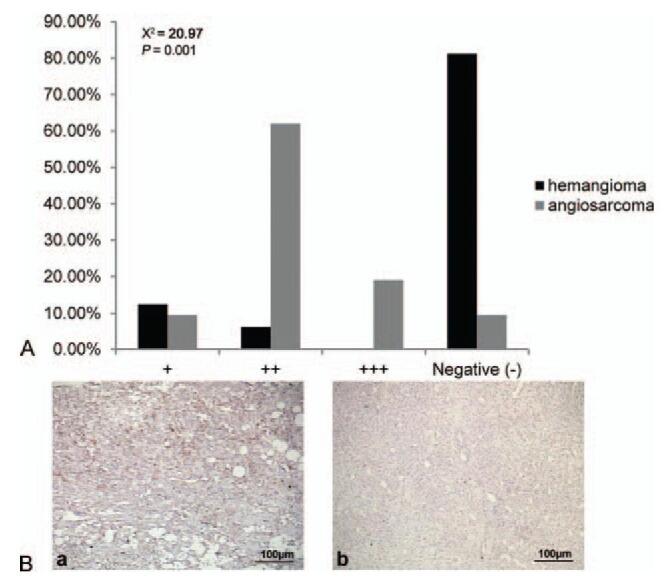
(A) PIK3CA immunoreactivity in angiosarcoma of the scalp and face and hemangioma. Angiosarcoma lesions exhibited significantly higher PIK3CA immunoreactivity than hemangioma (χ^2^ = 20.97, *P* = .001, *P* < .01). (B) Representative images of immunohistochemical staining of PIK3CA in (a) angiosarcoma and (b) hemangioma tissues (SP, ×100). PIK3CA = phosphatidylinositol-4,5-bisphosphate 3-kinase catalytic subunit alpha.

PIK3CA was exclusively detected in the cytoplasm. Angiosarcoma lesions exhibited significantly higher PIK3CA immunoreactivity than hemangiomas (χ^2^ = 20.97, *P* = .001, Fig. 3).

### 3.4 pMAPK was upregulated in scalp and face angiosarcoma

**Figure 4:**
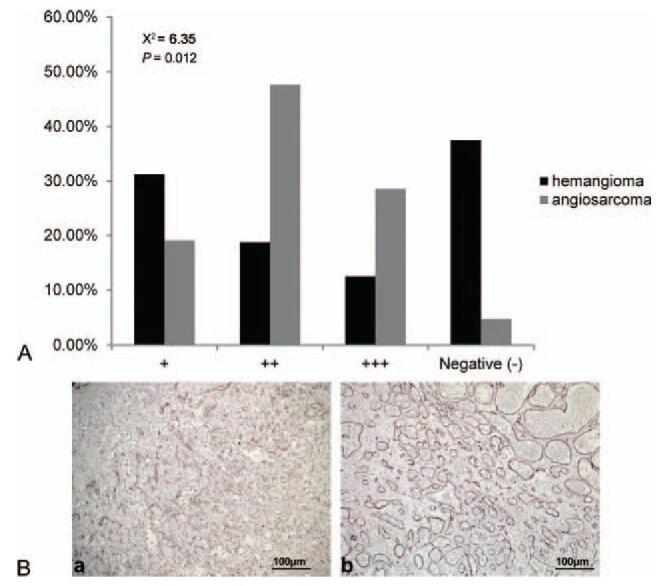
(A) pMARK immunoreactivity in angiosarcoma of the scalp and face and hemangioma. Angiosarcoma lesions exhibited significantly higher pMAPK immunoreactivity than hemangioma (χ^2^ = 6.35, *P* = .012, *P* < .05). (B) Representative images of immunohistochemical staining of pMAPK in (a) angiosarcoma and (b) hemangioma tissues (SP, ×100). pMAPK = phosphorylated mitogen activated kinase-like protein.

pMAPK was exclusively detected in the cytoplasm. Angiosarcoma lesions exhibited significantly higher pMAPK immunoreactivity than hemangiomas (χ^2^ = 6.35, *P* = .012, Fig. 4).

### 3.5 TP53 was upregulated in scalp and face angiosarcoma

**Figure 5:**
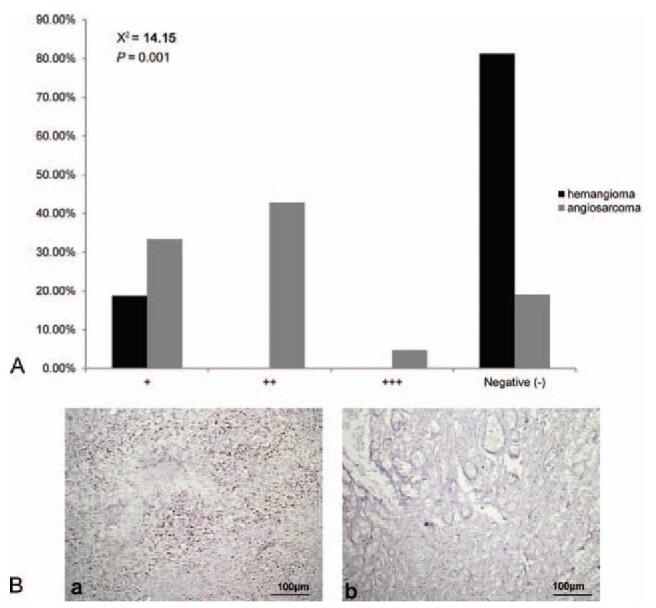
(A) TP53 immunoreactivity in angiosarcoma of the scalp and face and hemangioma. Angiosarcoma lesions exhibited significantly higher TP53 immunoreactivity than hemangioma (χ^2^ = 14.15, *P* = .001, *P* < .01). (B) Representative images of immunohistochemical staining of TP53 protein in (a) angiosarcoma and (b) hemangioma tissues (SP, ×100). TP53 = tumor protein p53.

TP53 was detected exclusively in the nucleus. Angiosarcoma lesions exhibited significantly higher TP53 immunoreactivity than hemangiomas (χ^2^ = 14.15, *P* = .001, Figure 5).

### 3.6 Scalp and face angiosarcoma showed decreased apoptosis than hemangioma

**Figure 6:**
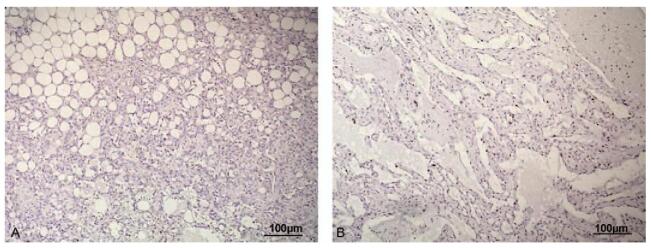
Representative images of TUNEL staining of angiosarcoma (A) and hemangioma (B) tissues (×100). Angiosarcoma lesions exhibited a significantly lower AI than those of hemangioma. AI = apoptosis index, TUNEL = transferase-mediated dUTP nick end-labeling.

Apoptotic nuclei showed a diffuse distribution pattern in both angiosarcoma and hemangioma tissues. They were mostly detected in tumor cells. Angiosarcoma lesions exhibited a significantly lower AI than hemangioma (12.16 ± 2.27 vs 21.17 ± 2.78, *t* = −4.532, *P* < .05, Fig. 6).

### 3.7 Correlation between PIK3CA, pMAPK, and TP53 and tumor differentiation

In angiosarcoma, the positive expression rates of PIK3CA, pMAPK, and TP53 were correlated with the degree of tumor differentiation (all *P* < .05) (Table 2). Only 3 cases were positive for PTEN, which could not be analyzed.

**Table 2:** PIK3CA, pMAPK, and TP53 expression in angiosarcoma with different degrees of tumor differentiation.

	Degree of tumor differentiation				
Gene expression	High (n = 7)	Moderate (n = 10)	Low (n = 4)	χ^2^	*P*
PIK3CA				13.338	.003
−	2	0	0		
+	2	0	0		
++	3	9	1		
+++	0	1	3		
PMAPK				13.978	.004
−	1	0	0		
+	3	1	0		
++	3	7	0		
+++	0	2	4		
P53				11.529	.023
−	3	0	0		
+	3	2	1		
++	1	8	2		
+++	0	0	1		

## 4 Discussion

In the present study, we detected significantly lower PTEN expression but higher PIK3CA, pMAPK, and TP53 expression in tumor tissues of scalp and face angiosarcoma than in benign hemangioma, and these differences in the expression of cancer-related proteins were correlated with the degree of tumor differentiation and coincided with reduced apoptosis in angiosarcoma compared to hemangioma.

The rarity of angiosarcoma makes large genomic studies to identify pathologic driver mutations almost impossible. Nonetheless, recent sequencing studies have implicated the PI3K, MAPK, and TP53 pathways as key oncogenic mechanisms driving the development of angiosarcoma.^[6–10]^ In 2019, Cao et al^[21]^ detected somatic alterations in PIK3CA and PIK3R1 (the 85 kDa regulatory subunit of PI3K) in the tumor specimen of a 51-year-old female patient with primary splenic angiosarcoma. In the angiosarcoma project,^[6]^ whole-exome sequencing of 47 human angiosarcoma specimens revealed recurrent PIK3CA and TP53 mutations, and scalp and face angiosarcoma was associated with a particularly high mutation burden. In a comprehensive genomic analysis of 34 cutaneous angiosarcomas using a sequencing assay for 341 known cancer-related genes, Murali et al^[10]^ found that over half of the tumors (18 out of 34) harbored genetic mutations or amplification of the MAPK pathway. In the same study, TP53 mutations were detected in 35% of cutaneous tumors (12 out of 34). Other investigations have also reported frequent TP53 mutations in human angiosarcomas.^[7–9]^ Collectively, these genetic studies strongly suggest that the PI3K, MAPK, and TP53 pathways play an important role in the development of angiosarcoma. In addition, Chadwick et al reported that PTEN was largely absent in high-grade angiosarcomas, while pMAPK was activated in all tumors of vascular origin.^[22]^ These results were in agreement with our findings that PTEN was downregulated and pMAPK was upregulated in scalp and face angiosarcoma compared with hemangioma. Of note, although bone angiosarcomas showed decreased expression of PTEN, PIK3CA hotspot mutations were absent, and no overexpression of TP53 was found in these tumors,^[23]^ suggesting that the driving mechanisms for angiosarcoma may depend on the location of the tumor.

The correlation between gene mutations and protein expression is very diverse. The mutation type was the most important determinant of expression levels.^[24]^ Although previous studies have reported mutations in the TP53,^[6–9]^ PI3K,^[6]^ and MAPK ^[10]^ pathways in angiosarcoma, these studies were genomic screening studies that did not examine protein levels. However, the present study only examined protein expression and did not examine the mutations. In addition, a wide variety of mutations involved in malignant transformation were not possible in the present study. TP53 is a tumor suppressor gene, and its downregulation or mutational inactivation is associated with cancer.^[25]^ PI3K is a signal transducer that activates the AKT pathway, which plays multiple roles in cell proliferation, survival, and motility.^[17]^ MAPK is also a signal transducer that participates in cell proliferation, survival, and motility.^[16]^ Mutations that constitutively these 2 proteins would lead to malignant transformation,^[16,17]^ and constitutive overexpression of these 2 proteins would achieve the same end.^[26–29]^ Future studies should examine the mutation burden along with protein expression in the scalp and face angiosarcoma.

Several functional studies have provided preliminary evidence supporting the PD-1/PD-L1, PI3K, and mammalian target of rapamycin (mTOR) pathways as therapeutic targets for cutaneous angiosarcoma. The PD-1/PD-L1 pathway regulates the induction of immune tolerance in the tumor microenvironment. The expression of PD-L1 in tumor cells has been shown to stimulate aerobic glycolysis by activating the PI3K-AKT-mTOR pathway, which directly affects tumor cell metabolism.^[30]^ Aberrant expression of PD-1 has been detected in a number of angiosarcomas at different body locations,^[31]^ and treating a patient with metastatic cutaneous angiosarcoma with the anti-PD-1 monoclonal antibody pembrolizumab resulted in almost complete eradication of the metastatic lesions.^[32]^ The PI3K/Akt/mTOR pathway has been known to be frequently dysregulated in breast cancer and other types of cancers and therefore has been identified as an important target in breast cancer research. First-generation inhibitors of mTOR have been approved for the treatment of breast, pancreatic, renal, and some brain cancers. Moreover, some second-generation mTOR inhibitors and novel agents targeting PI3K or Akt have been identified in early clinical trials. One study found activating mutations in PIK3CA in 40% of tumors, while other investigators described elevated levels of phosphorylated S6 in 100% and phosphorylated 4EBP1 in 88% of angiosarcomas, both of which are downstream effectors of mTOR.^[17]^ Importantly, 1 study demonstrated that treatment with the mTOR inhibitor rapamycin decreased cell proliferation in vitro and delayed tumor growth in vivo using a xenograft model.^[33]^ The PI3K inhibitor LY294002 and mTOR inhibitor everolimus (RAD001) inhibited the growth of cutaneous angiosarcoma cells by inducing G1 cell cycle arrest.^[34]^ Moreover, the knockdown of PDK1, the first node of the PI3K signal output, inhibited colony formation in cutaneous angiosarcoma cells.^[34]^ These and future proof-of-concept studies might guide the development of targeted therapies for angiosarcoma.

The current study had some limitations. The sample size was small and was from a single center. Angiosarcomas are very rare, accounting for approximately 1% to 2% of soft tissue sarcomas,^[35]^ which represent only 1% to 2% of all tumors.^[36]^ We included all cases that were identified at our hospital. In addition, the possibility of differential diagnoses in angiosarcoma patients could not be ruled out; thus, some cases were not identified in this database. The protein markers involved in this study were mainly related to the PD-1/PD-L1 and PI3K/mTOR signaling pathways. The pathogenesis of angiosarcoma is probably also related to other genes, including CIC gene rearrangement,^[37]^ increased expression of the *MYC* gene^[38]^ and *FLT-4* gene,^[37]^ and mutations of *PTPRB* and *PLCG1*,^[1,39–43]^ but the present study focused on the PD-1/PD-L1 and PI3K/mTOR signaling pathways. Many patients visited our hospital for diagnosis and/or initial treatment and were sent back to their local hospital. Therefore, the prognosis cannot be determined. Further pathway studies are needed to clarify the mechanisms of angiosarcoma.

## 5 Conclusion

In summary, we detected aberrant PTEN, PIK3CA, pMAPK, and TP53 expression and reduced tumor cell apoptosis in human angiosarcoma of the scalp and face compared with hemangioma, suggesting that the PI3K, MAPK, and TP53 pathways might be associated with angiosarcoma tumorigenesis and the degree of tumor differentiation. PTEN, PIK3CA, pMAPK, and TP53 may serve as therapeutic targets for the effective treatment of this rare and aggressive malignancy.

## Acknowledgments

We are grateful to all patients for supporting the investigation.

## Author contributions

**Conceptualization:** Dingding Zhang.

**Data curation:** Huiying Wan.

**Formal analysis:** Dingding Zhang, Weimin Hu, Zhen Xie.

**Funding acquisition:** Dingding Zhang.

**Investigation:** Qiu Du.

**Methodology:** Qiu Du, Qiongrong Xia, Taishen Wen, Haiping Jia.

**Revised the manuscript:** Dingding Zhang.

**Writing – original draft:** Huiying Wan.

**Writing – review & editing:** Dingding Zhang.
